# Impact on Glycemia Risk Index and other metrics in type 1 adult patients switching to Advanced Hybrid Closed-Loop systems: a one-year real-life experience

**DOI:** 10.1186/s40001-024-01946-w

**Published:** 2024-07-15

**Authors:** Eugenia Resmini, Emanuela Zarra, Silvia Dotti, Giulia Rotondi, Angelo Vincenzo Cornaghi, Sara Madaschi, Elena Cimino, Giulia Massari, Letizia Chiara Pezzaioli, Caterina Buoso, Marco Sandri, Angela Girelli

**Affiliations:** Medicina Generale Diabetologia, Dipartimento di Continuità di Cura e Fragilità, ASST Spedali Civili, Brescia, Italy

**Keywords:** Advanced Hybrid Closed-Loop systems, GRI, CGM metrics, Type 1 diabetes mellitus

## Abstract

**Background:**

Advanced Hybrid Closed-Loop system (AHCL) has profoundly changed type 1 diabetes therapy. This study primarily aimed to assess the impact on Glycemia Risk Index (GRI) and other continuous glucose monitoring (CGM) metrics when switching from one of four insulin strategies to AHCL in type 1 adult patients.

**Methods:**

A single-center, retrospective pre/post observational study; 198 patients (age 44.4 ± 12.7 years, 115 females/83 males, diabetes duration 24.7 ± 11.6 years, HbA1c 7.4 ± 1%), treated with different insulin therapies (MDI, CSII, SAP with PLGS, HCL) were assessed before and after switching to an AHCL (MiniMed 780G, Diabeloop Roche, Tandem Control-IQ) at 1, 3, 6, and 12 months. Mixed-effects multivariable regression models were used to estimate the mean pre/post variations at different time points, adjusted for potential confounders.

**Results:**

A month after the switch, there was an improvement in CGM metrics and HbA1c for all patients: GRI −10.7, GMI −0.27%, CV −2.1%, TAR_>250_ −3.7%, TAR_180-250_ −5.6%, TIR + 9.7%, HbA1c −0.54% (all p < 0.001). This improvement was maintained throughout the observational period (at 3, 6, and 12 months, with all p-values < 0.001). When improvements across the 780, Diabeloop, and Tandem CIQ devices were compared: Diabeloop demonstrated significantly better performance in terms of GRI, GMI, CV, TAR_>250_ at T1 (for all p < 0.01); 780 recorded highest average decrease in TAR_180-250_ (p = 0.020), while Tandem achieved the most significant reduction in TBR_54-69_ (p = 0.004).

**Conclusions:**

Adopting an AHCL leads to a rapid and sustained improvement in GRI and other parameters of metabolic control for up to a year, regardless of prior insulin therapies, baseline conditions or brands.

**Supplementary Information:**

The online version contains supplementary material available at 10.1186/s40001-024-01946-w.

## Introduction

The treatment of diabetes, in particular that of type 1 diabetes, has deeply changed due to the increasingly use of technologies both for continuous glucose monitoring (CGM), and for continuous insulin infusion (Continuous Subcutaneous Insulin Infusion, CSII) and their integration through the use of automatic systems equipped with algorithms (Advance Hybrid Closed Loop, AHCL) [1–4]. Therefore, the use of sensor metric parameters, which provide much more precise information on metabolic trends (not just blood glucose assessment mean and standard deviation but evaluation of glycemic variability in terms of coefficient of variation, Time in Range, Time above Range, Time Below Range) has been added to glycated hemoglobin (HbA1c) [5–8].

The Glycemia Risk Index (GRI) is novel metric that in a single-number summarizes the global glycemic effects [9, 10]. The GRI is a risk score that combines hypoglycemia and hyperglycemia risk into a single number as a percentile from 0 (the lowest risk) to 100 (the highest risk), but TBR_54-69_ and TBR_<54_ have a greater weight than TAR_>250_ and TAR_180-250._ Hence, the risk of hypoglycemia based on TBR is represented more visibly in GRI.

Additionally, TAR_>250_ and TBR_<54_ have a higher weight than TAR_180-250_ and TBR_54-69_, respectively. Thus, improvement in severe hypoglycemia and hyperglycemia is represented more prominently with the GRI [5].

A recent consensus added GRI to define glycemic control derived from CGM [11]. The GRI is not routinary used to date, few data on its role are recently emerging in a study on Tandem Control IQ [12] on MiniMed 780G [13] and on Diabeloop Roche [14].

The routine introduction of CGM systems has also determined a profound modification of the evaluation of clinical outcomes; although the optimum use “basal-bolus” insulin therapy, the effectively reduction of hypoglycemic risk, glycemic variability, glycemic targets is often not reached. The achievement of glycemic control goals in type 1 diabetes mellitus (T1D) is inextricably linked to a significant reduction in the onset and progression of microvascular complications, such as diabetic retinopathy, nephropathy, and neuropathy [15–17].

The insight gained from ADAPT study [18], combined with that from previously published studies of other automated insulin delivery systems, suggests that AHCL should be considered at the early stages in the type 1 diabetes treatment pathway, in order to have reduced risk of long-term complications [18]. Moreover, the combined benefits in terms of HbA1c, time-in-range, patient reported outcomes, and the potential long-term implications of this further suggest that AHCL should be considered early in the course of the disease when the use of multiple daily injections of insulin plus is CGM fails to achieve targets [16]

The three main AHCL systems currently available in Italy are: MiniMed 780G, Diabeloop Roche, Tandem Control-IQ [19], which on average reduce the TIR in a similar way but use different algorithms and different tools.

This study primarily aimed to assess the impact on Glycemia Risk Index (GRI) and other CGM metrics when switching from one of four insulin strategies to AHCL in type 1 adult patients, in a real-world context.

## Methods

### Study design and patients

A single-center, retrospective pre/post observational study was conducted on a cohort of diabetic patients followed by the Diabetology Department of Spedali Civili di Brescia. Data were obtained from the diabetic electronic record of the Department (FenixAmb^®^ by EL.CO.), in patients who switched to an AHCL. The observation period was from November 1st, 2020, to March 31st, 2023. A total of 198 patients with type 1 DM have been included in this study (age 44.4 ± 12.7 years, 115 females/83 males, BMI 25.7 ± 4.7, diabetes duration 24.7 ± 11.6 years, HbA1c 7.4 ± 1%).

At baseline, the patients were treated with one of four different insulin therapies:

- Multiple daily injections (MDI) of insulin;
- Continuous subcutaneous insulin infusion (CSII) with an associated sensor;
- Sensor-augmented pump (SAP) with predictive low-glucose suspend (PLGS) and with low glucose suspend (LGS), (Minimed™640G, Medtronic, Northridge, CA, Basal IQ);
- Hybrid closed loop (HCL) system (Minimed™670G, Medtronic, Northridge, CA).

The patients were assessed before and after switching to an AHCL system (Minimed 780G, Diabeloop Roche, Tandem Control-IQ) at 1, 3, 6, and 12 months. Pre-switch data were obtained at 1, 3, 6 months.

The inclusion criteria were: type 1 DM duration for at least one year, C peptide < 0.3 ng/mL, current insulin treatment for at least 6 months, age between 18 and 70 years, a signed informed consent form.

The exclusion criteria were: patients who did not have a post-switch observation period of at least 12 months, patients who did not have a pre-switch observation period of at least 6 months, pregnant women, patients who had not uploaded data from their continuous glucose monitoring platforms.

The study was approved from the Local Ethic Committee of Spedali Civili di Brescia (protocol number 5828).

All patients were already using carbohydrate counting and wearing a glucose sensor for over 70% of the time. Of these, 48 (24%) patients were utilizing flash glucose monitoring (FGM) and 150 (76%) continuous glucose monitoring (CGM).

The participants received individual educational sessions on the features of the AHCL system and if necessary, they also had online interactions and/or phone calls with the diabetes team.

### Outcomes

The primary outcome of this study was to assess the impact on GRI when switching from one of four insulin strategies to AHCL in type 1 adult patients.

Secondary outcomes were:

- To assess the impact on other continuous glucose monitoring (CGM) metrics and HbA1c after the switch.
- To compare the different AHCL devices in order to identify any differences in terms of metabolic effectiveness.
- To verify whether there was an association between the use of these technologies and the incidence of acute complications and the incidence/progression of chronic complications in the observation period. Data were obtained from the diabetic electronic record of the Department (FenixAmb^®^ by EL.CO.).

### Statistical analyses

Continuous variables were summarized as mean and standard deviation (SD), while categorical variables were described in terms of absolute and relative frequencies. To compare means and proportions among three or more patient subgroups, the Kruskal–Wallis test and Fisher’s exact test were employed, respectively.

Given the longitudinal design of the study, mixed-effects multivariable models were employed to estimate and compare the average pre- and post-intervention changes of HbA1c and CGM metrics at the study timepoints, while controlling for potential confounders. Mixed-effects regression is a statistical method that accounts for within-subject correlation and allows the explicit modelling of a variety of correlation patterns [20]. The parameters were estimated using the restricted maximum likelihood, applying the modification proposed by Kenward and Roger to account for limited sample size [21].

When estimating the pre/post variation for each outcome across the entire cohort, the mixed-effects model included a random effect for the intercept (attributable to patients), the timepoint variable, and a set of adjustment variables (age, gender, disease duration, and previous insulin strategies).

We used Spearman’s rank correlation coefficient to investigate the (monotonic) association between variables measured at the same timepoint.

To compare the pre/post variations of each outcome between subgroups, a (time x subgroup) interaction term was introduced in the model. The statistical significance of the interaction term was tested using a likelihood ratio test; a p-value of less than 0.05 indicates significant differences in pre/post variations among subgroups.

Statistical analyses were performed using Stata 16.1 (StataCorp. College Station, TX, USA) and R 4.3.1 (R Foundation for Statistical Computing, Vienna, Austria).

## Results

A total of 198 patients switched to AHCL therapy, 99 (50%) patients to Minimed 780G, 16 (8%) to Diabeloop and 83 (42%) to Tandem CIQ.

Among these 99 using Minimed 780G, 6 came from traditional CSII, 10 from MDI, 41 from SAP and 42 from HCL. Of the 16 using Diabeloop, 15 came from CSII and 1 from HCL. Of the 83 using Tandem CIQ, 38 came from CSII, 10 from MDI, 34 from SAP and 1 from HCL. These data are shown in Table 1.

**Table 1:** Baseline clinical and metabolic characteristics for the entire study cohort and stratified by AHCL systems (780, DIABELOOP, TANDEM CIQ)

Parameters	All patients (N = 198)	780 (N = 99)	DIABELOOP (N = 16)	TANDEM CIQ (N = 83)	P
Age (years)	44.4 ± 12.7	45.6 ± 11.9	44.6 ± 12.7	43 ± 13.6	0.29
Sex (female)	115 (58.1)	48 (48.5)	12 (75)	55 (66.3)	0.019
Duration of the disease (years)	24.7 ± 11.6	23.8 ± 11.2	28.3 ± 12.3	25.2 ± 11.8	0.4
BMI (Kg/m^2^)	25.7 ± 4.7	26 ± 5.2	27 ± 3.6	25.1 ± 4.2	0.2
HbA1c (%)	7.4 ± 1	7.4 ± 0.9	7.9 ± 1.2	7.4 ± 1	0.3
Previous therapy					< 0.001
Traditional CSII with associated sensor	59 (29.8)	6 (6.1)	15 (93.8)	38 (45.8)	
MDI	20 (10.1)	10 (10.1)	0 (0)	10 (12)	
SAP plus LGS and PLGS	75 (37.9)	41 (41.4)	0 (0)	34 (41)	
HCL	44 (22.2)	42 (42.4)	1 (6.2)	1 (1.2)	
Age of onset for diabetes (years)	19.7 ± 12.5	21.8 ± 13.8	16.3 ± 7.7	17.8 ± 11.2	0.1
Duration of the previous therapy (months)	32.7 ± 38.1	37.3 ± 34.2	50.1 ± 29.8	23.3 ± 42.3	< 0.001

CSII, Continuous subcutaneous insulin infusion; MDI, Multiple daily injections; SAP, Sensor-Augmented Pump; LGS, Low Glucose Suspend; PLGS, Predictive Low Glucose Suspend; HCL, Hybrid Closed-Loop; BMI, body mass index; GRI, glycaemia risk index; CV, Coefficient of variation; GMI, Glucose management index; F, female; FGM, Flash glucose monitoring; TAR, Time above range; TBR, Time below range; TIR, Time in rage

Continuous variables are presented as mean ± standard deviation; categorical variables as absolute and relative frequencies

Table 2 summarizes the observed values of HbA1c and CGM metrics at different time points for the entire study cohort, along with the percentage of patients simultaneously achieving the targets of HbA1c < 7%, TIR > 70%, and TBR < 4%.

**Table 2:** Mean values and standard deviations of HBA1c and CGM metrics at all the study timepoints and for the entire study cohort

Parameters	**T = -6**	**T = -3**	**T = -1**	**T = 0**	**T = 1**	**T = 3**	**T = 6**	**T = 12**	**P**
All subjects (N = 198)									
HbA1C (%)	7.4 ± 0.9	7.4 ± 0.9	7.4 ± 0.9	7.4 ± 1	6.8 ± 0.8	6.8 ± 0.8	6.8 ± 0.8	6.9 ± 0.8	< 0.001
GRI	40.2 ± 22.1	39 ± 21.1	39.2 ± 20.9	37.5 ± 18.4	26.8 ± 13	26.9 ± 14.1	27.6 ± 14.8	28.2 ± 14.6	< 0.001
GMI	7.1 ± 0.7	7.1 ± 0.7	7.1 ± 0.6	7.1 ± 0.6	6.8 ± 0.5	6.8 ± 0.5	6.8 ± 0.5	6.9 ± 0.5	< 0.001
CV	34.8 ± 7	34.3 ± 5.9	34.8 ± 6.4	34.6 ± 6.1	32.4 ± 5.3	32.2 ± 5.1	32.8 ± 5.4	32.7 ± 5.5	< 0.001
TAR_>250_ (%)	8.4 ± 10	8.6 ± 10.4	8.9 ± 9.8	8.1 ± 9.7	4.4 ± 5.7	4.7 ± 6	4.9 ± 6.7	5.5 ± 6.7	< 0.001
TAR_180-250_ (%)	22.5 ± 9.4	22.6 ± 9.6	22.1 ± 9.3	22.2 ± 8.9	16.8 ± 7.6	16.9 ± 7.9	17.3 ± 7.8	17.6 ± 7.6	< 0.001
TIR %	65.6 ± 17.2	66 ± 16.6	66.1 ± 16.3	66.8 ± 15.9	76.3 ± 11.2	76.1 ± 12	75.5 ± 12.5	74.8 ± 12.3	< 0.001
TBR_54-69_ (%)	2.7 ± 2.6	2.2 ± 2	2.2 ± 1.9	2.3 ± 2.1	2 ± 1.7	1.8 ± 1.7	1.9 ± 1.6	1.6 ± 1.5	< 0.001
TBR_<54_ (%)	0.8 ± 1.8	0.7 ± 1.3	0.6 ± 1.2	0.6 ± 0.9	0.5 ± 0.8	0.5 ± 0.8	0.5 ± 0.8	0.5 ± 0.7	0.1
HbA1c < 7%, TIR > 70%, TBR < 4%	17.3%	15.7%	17.4%	12.5%	41.5%	40.9%	42.7%	43.8%	< 0.001

P-values reported in the P column are obtained from the likelihood ratio test applied to the multivariable mixed-effects models for the comparison of mean values across all study time points

### Overall pre/post differences

A month after the switch (T1 timepoint), there was a significant improvement in CGM metrics and HbA1c for all patients, even after adjusting for age, gender, disease duration, and previous insulin strategies: GRI −10.7, GMI −0.27%, CV −2.1%, TAR_>250_ −3.7%, TAR_180-250_ −5.6%, TIR + 9.7%, HbA1c −0.54% (all p < 0.001, Table 3).

The improvements observed after the switch were maintained throughout the observational period. At 3 months following the switch, significant improvements were observed: GRI −10.7, GMI −0.25%, CV −2.3%, TAR_>250_ −3.2%, TAR_180-250_ −5.4%, TIR + 9.2%, HbA1c −0.55% (all p < 0.001). At T3, the variation in TBR_54-69_ also became significant (−0.43%, p = 0.007). These improvements persisted at 6 months: GRI −9.5, GMI −0.22%, CV −1.6%, TAR_>250_ −3.2%, TAR_180-250_ −4.9%, TIR + 8.5%, HbA1c −0.57% (all p < 0.001), and TBR_54-69_ −0.37% (p = 0.02). At 12 months, the favourable trends continued: GRI −9, GMI −0.18%, CV −1.6%, TAR_>250_ −2.6%, TAR_180-250_ −4.6%, TIR + 7.9%, HbA1c −0.52%, and TBR_54-69_ −0.62% (all p < 0.001). For TBR_<54_, no significant improvements were detected at any timepoint.

No significant gender differences have been evidenced. BMI did not significantly change after the switch. At the T0 timepoint, the percentage of overweight and obese females was 29.5% and 15.2%, respectively; these percentages did not change significantly at T12 (26.1% and 16.2%, respectively). For males, the percentages of overweight and obese individuals were 44.4% and 17.3% at T0, and 46% and 13.5% at T12 (no statistically significant change from T0 to T12). At T0 and T12, the weight of females was 66.6 ± 14.5 kg and 67.3 ± 15.4 kg, respectively (p = 0.12), while for males it was 81.9 ± 13.9 kg and 81.1 ± 13.3 kg (p = 0.54).

An analysis of the association between GRI and CGM metrics revealed a strong and statistically significant association at T3 and T6 for: GRI and TAR_>250_ (R = 0.86, p < 0.001), GRI and TAR_180-250_ (R = 0.77, p < 0.001), and GRI and TIR (R = -0.94, p < 0.001). However, no significant association emerged between GRI and TBR_54-69_ or TBR_<54_ (with R values of 0.09, p = 0.22, and 0.31, p < 0.001, respectively).

**Fig. 1:**
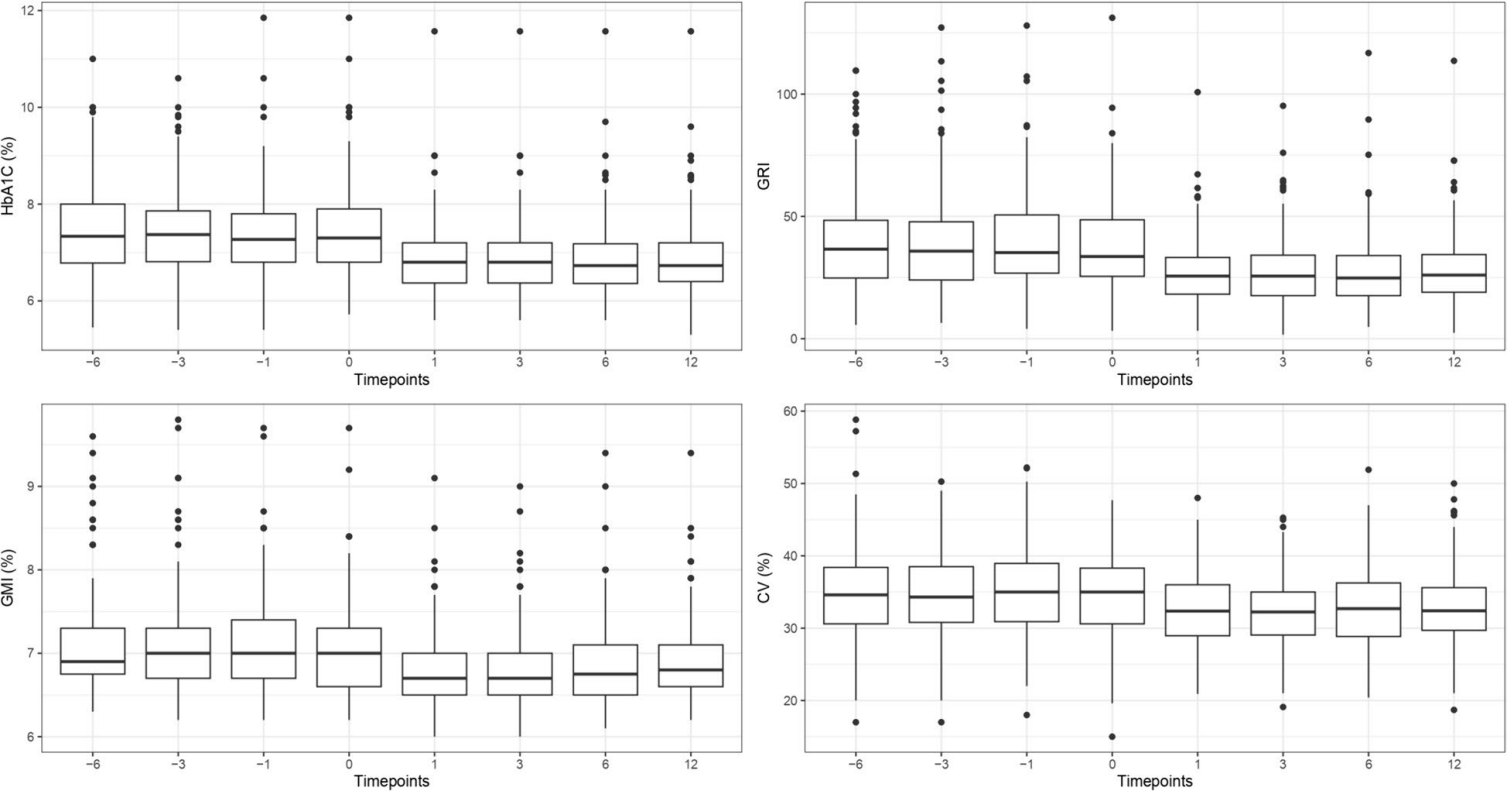
Boxplot showing the distribution of HBA1c, GRI, GMI, CV in all patients, over the entire study period

**Fig. 2:**
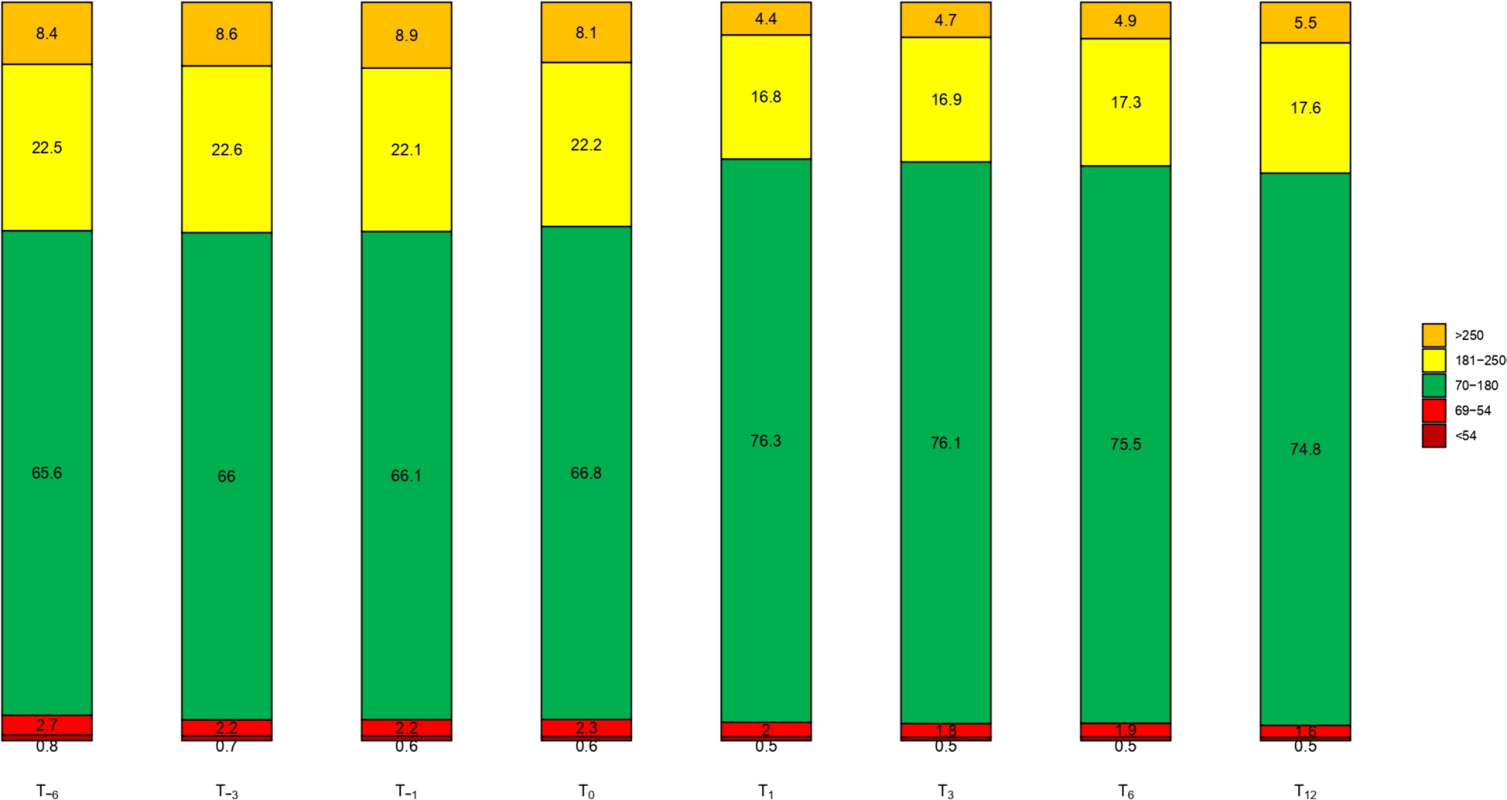
TAR, TIR, TBR in all patients over the entire study period

Figure 1 shows the statistical distribution of HbA1c, GRI, GMI, and CV at the study time points for the entire cohort, using boxplots. Figure 2 illustrates the percentages for TAR, TIR, and TBR with the barplot. These visualizations provide a comprehensive overview of the key metrics and their variations over the study period.

### Pre/post differences by AHCL groups

Table 3 summarizes the variations in CGM metrics and HbA1c after the switch, comparing the Minimed 780G, Diabeloop, and Tandem CIQ devices at the T1, T3, T6, and T12 time points (1, 3, 6, and 12 months).

**Table 3:** Mean variations in HbA1c and CGM metrics post-therapy switch (with 95% CIs), for the entire cohort and by AHCL therapy

Parameter	Comparison	All patients (N = 198)	780 (N = 99)	Diabeloop (N = 16)	Tandem (N = 83)	P
HbA1c (%)	T0 vs T1	−0.54 (−0.65 to −0.44)^***^	−0.47 (−0.6 to −0.35)^***^	−0.84 (−1.3 to −0.35)^**^	−0.58 (−0.77 to −0.39)^***^	0.2
	T0 vs T3	−0.55 (−0.66 to −0.44)^***^	−0.48 (−0.6 to −0.35)^***^	−0.84 (−1.3 to −0.35)^**^	−0.6 (−0.78 to −0.41)^***^	0.2
	T0 vs T6	−0.57 (−0.67 to −0.47)^***^	−0.49 (−0.61 to −0.37)^***^	−0.9 (−1.31 to −0.48)^***^	−0.61 (−0.79 to −0.42)^***^	0.1
	T0 vs T12	−0.52 (−0.62 to −0.41)^***^	−0.42 (−0.54 to −0.31)^***^	−0.78 (−1.07 to −0.49)^***^	−0.59 (−0.8 to −0.39)^***^	0.1
GRI	T0 vs T1	−10.7 (−12.8 to −8.6)^***^	−9 (−11.6 to −6.4)^***^	−20.6 (−32.3 to −8.9)^**^	−11.1 (−14.5 to −7.8)^***^	0.032
	T0 vs T3	−10.7 (−12.8 to −8.5)^***^	−9.8 (−12.5 to −7)^***^	−14.9 (−24.6 to −5.2)^**^	−11 (−14.5 to −7.5)^***^	0.5
	T0 vs T6	−9.5 (−11.5 to −7.5)^***^	−9.1 (−12.1 to −6.2)^***^	−13.4 (−21.3 to −5.6)^**^	−9.4 (−12.3 to −6.4)^***^	0.6
	T0 vs T12	−9 (−11 to−7.1)^***^	−8.5 (−11.3 to −5.8)^***^	−11.3 (−18.5 to −4.1)^**^	−9.2 (−12.2 to −6.3)^***^	0.8
GMI (%)	T0 vs T1	−0.27 (−0.33 to −0.20)^***^	−0.36 (−0.43 to −0.28)^***^	−0.47 (−1.04 to 0.11)	−0.17 (−0.26 to −0.07)^**^	0.008
	T0 vs T3	−0.25 (−0.31 to −0.18)^***^	−0.31 (−0.39 to −0.22)^***^	−0.4 (−0.83 to 0.03)	−0.18 (−0.27 to −0.09)^***^	0.1
	T0 vs T6	−0.22 (−0.28 to −0.15)^***^	−0.29 (−0.38 to −0.2)^***^	−0.31 (−0.65 to 0.02)	−0.14 (−0.24 to −0.05)^**^	0.1
	T0 vs T12	−0.18 (−0.24 to −0.11)^***^	−0.23 (−0.31 to −0.14)^***^	−0.3 (−0.62 to 0.02)	−0.12 (−0.22 to −0.02)^*^	0.2
CV (%)	T0 vs T1	−2.1 (−2.9 to −1.2)^***^	−0.7 (−1.9 to 0.4)	−7.3 (−10.5 to −4)^***^	−2.7 (−3.9 to −1.5)^***^	< 0.001
	T0 vs T3	−2.3 (−3.1 to −1.4)^***^	−1.1 (−2.2 to 0.1)	−6.8 (−10.4 to −3.3)^***^	−2.8 (−4 to −1.6)^***^	0.002
	T0 vs T6	−1.6 (−2.5 to −0.7)^***^	−0.4 (−1.6 to 0.8)	−5.9 (−9.8 to −2.1)^**^	−2.2 (−3.5 to −1)^**^	0.005
	T0 vs T12	−1.6 (−2.4 to −0.8)^***^	−1.1 (−2.4 to 0.1)	−4.9 (−7.1 to −2.6)^***^	−1.6 (−2.9 to −0.4)^**^	0.1
TAR_>250_ (%)	T0 vs T1	−3.7 (−4.9 to −2.5)^***^	−2.5 (−3.4 to −1.7)^***^	−9.5 (−16.8 to −2.1)^*^	−4.2 (−6.5 to −1.9)^***^	0.017
	T0 vs T3	−3.2 (−4.3 to −2.2)^***^	−2 (−3 to −1.1)^***^	−7.5 (−13.8 to −1.2)^*^	−3.9 (−5.6 to −2.1)^***^	0.018
	T0 vs T6	−3.2 (−4.3 to −2)^***^	−2.3 (−3.3 to −1.3)^***^	−6 (−10.4 to −1.7)^**^	−3.6 (−5.7 to −1.5)^**^	0.2
	T0 vs T12	−2.6 (−3.6 to −1.5)^***^	−1.7 (−2.6 to −0.9)^***^	−5.7 (−10.3 to −1)^*^	−3 (−5.1 to −0.9)^**^	0.2
TAR_180−250_ (%)	T0 vs T1	−5.6 (−6.8 to −4.5)^***^	−7.2 (−9 to −5.5)^***^	−4.7 (−7.6 to −1.8)^**^	−4 (−5.6 to −2.5)^***^	0.020
	T0 vs T3	−5.4 (−6.5 to −4.3)^***^	−7 (−8.7 to −5.3)^***^	−3.2 (−5.4 to −1)^**^	−4.1 (−5.6 to −2.5)^***^	0.021
	T0 vs T6	−4.9 (−6 to −3.8)^***^	−6.4 (−8.2 to −4.7)^***^	−3.6 (−6.4 to −0.8)^**^	−3.6 (−5 to −2.2)^***^	0.037
	T0 vs T12	−4.6 (−5.8 to −3.4)^***^	−5.5 (−7.4 to −3.6)^***^	−2.9 (−6.5 to 0.6)	−3.8 (−5.3 to −2.3)^***^	0.3
TIR_70−180_ (%)	T0 vs T1	9.7 (7.8 to 11.5)^***^	9.5 (7.2 to 11.7)^***^	14.8 (6.7 to 22.8)^***^	9.2 (5.9 to 12.4)^***^	0.4
	T0 vs T3	9.2 (7.5 to 10.9)^***^	9.4 (7.1 to 11.8)^***^	11 (4.2 to 17.8)^**^	8.7 (6 to 11.4)^***^	0.8
	T0 vs T6	8.5 (6.7 to 10.3)^***^	8.8 (6.4 to 11.3)^***^	10 (4 to 16)^**^	8 (5.2 to 10.7)^***^	0.8
	T0 vs T12	7.9 (6.1 to 9.7)^***^	7.8 (5.4 to 10.2)^***^	8.6 (2.9 to 14.3)^**^	7.9 (4.9 to 10.8)^***^	1
TBR_54−69_ (%)	T0 vs T1	−0.26 (−0.56 to 0.04)	0.24 (−0.23 to 0.71)	−0.34 (−1.4 to 0.67)	−0.79 (−1.17 to −0.41)^***^	0.004
	T0 vs T3	−0.43 (−0.74 to 0.12)^**^	0.16 (−0.63 to 0.31)	−0.03 (−1.21 to 1.14)	−0.77 (−1.21 to −0.33)^**^	0.1
	T0 vs T6	−0.37 (−0.69 to −0.06)^*^	−0.09 (−0.6 to 0.42)	−0.26 (−1.48 to 0.96)	−0.69 (−1.07 to −0.3)^**^	0.2
	T0 vs T12	−0.62 (−0.93 to −0.32)^***^	−0.39 (−0.84 to 0.06)	0.03 (−1.07 to 1.13)	−0.97 (−1.38 to −0.55)^***^	0.1
TBR_<54_ (%)	T0 vs T1	−0.04 (−0.18 to 0.09)	0.11 (−0.1 to 0.32)	−0.22 (−0.56 to 0.12)	−0.19 (−0.39 to 0.01)	0.1
	T0 vs T3	−0.1 (−0.24 to 0.04)	−0.14 (−0.36 to 0.07)	−0.03 (−0.51 to 0.45)	−0.07 (−0.27 to 0.14)	0.8
	T0 vs T6	−0.07 (−0.22 to 0.07)	−0.01 (−0.23 to 0.22)	−0.13 (−0.59 to 0.34)	−0.14 (−0.34 to 0.07)	0.7
	T0 vs T12	−0.11 (−0.25 to 0.04)	−0.12 (−0.35 to 0.11)	0.01 (−0.38 to 0.39)	−0.11 (−0.29 to 0.07)	0.9

Pre/post variations as estimated using multivariable mixed-effect logistic regression analysis across four timepoints

Asterisks indicate the statistical significance of the pre/post variation according to the following: * p < 0.05, ** p < 0.01, and *** p < 0.001

P-values reported in the P column are obtained from the likelihood ratio test applied to the multivariable mixed-effects models for comparing mean pre/post variations across AHCL therapy groups

The comparison showed that at T1, the Diabeloop device significantly outperformed the others in terms of GRI, GMI, CV, TAR_>250_. Specifically, the Diabeloop group exhibited a mean GRI reduction of 20.6, compared to a reduction of 9 and 11.1 in the 780 and Tandem groups, respectively (P = 0.032). The GMI in the Diabeloop group decreased by 0.47%, versus 0.36% in the Minimed 780G group and 0.17% in the Tandem group (p = 0.008). Similarly, CV decreased by 7.3% with Diabeloop, in contrast to decreases of 0.7% and 2.7% in the 780 and Tandem groups, respectively (p < 0.001). The mean reduction in TAR_>250_ was 9.5% for Diabeloop, compared to 2.5% for 780 and 4.2% for Tandem (p = 0.017). Furthermore, at T1, the Minimed 780G device recorded the highest average decrease in TAR_180-250_ at 7.2% (compared to 4.7% for Diabeloop and 4% for Tandem, p = 0.020), while Tandem achieved the most significant reduction in TBR_54-69_ at −0.79 (+ 0.24 for 780 and −0.34 for Diabeloop, p = 0.004).

Beyond T1, the previously observed differences in performance improvements for GRI, GMI, TAR_>25_ and TBR_54-69_ across the three devices were no longer evident. Diabeloop continued to demonstrate statistically significant better CV reductions at the later assessments of T3 and T6 (p = 0.002 and p = 0.005, respectively) and a significantly better TAR_>250_ at T3 (p = 0.018). The superiority of the Minimed 780G device in reducing TAR_180-250_ continued to be evident at T3 and T6 (p = 0.021 and p = 0.037, respectively).

The HbA1c reductions achieved by the three devices evidenced no statistical differences at 1, 3, 6, and 12 months.

**Fig. 3:**
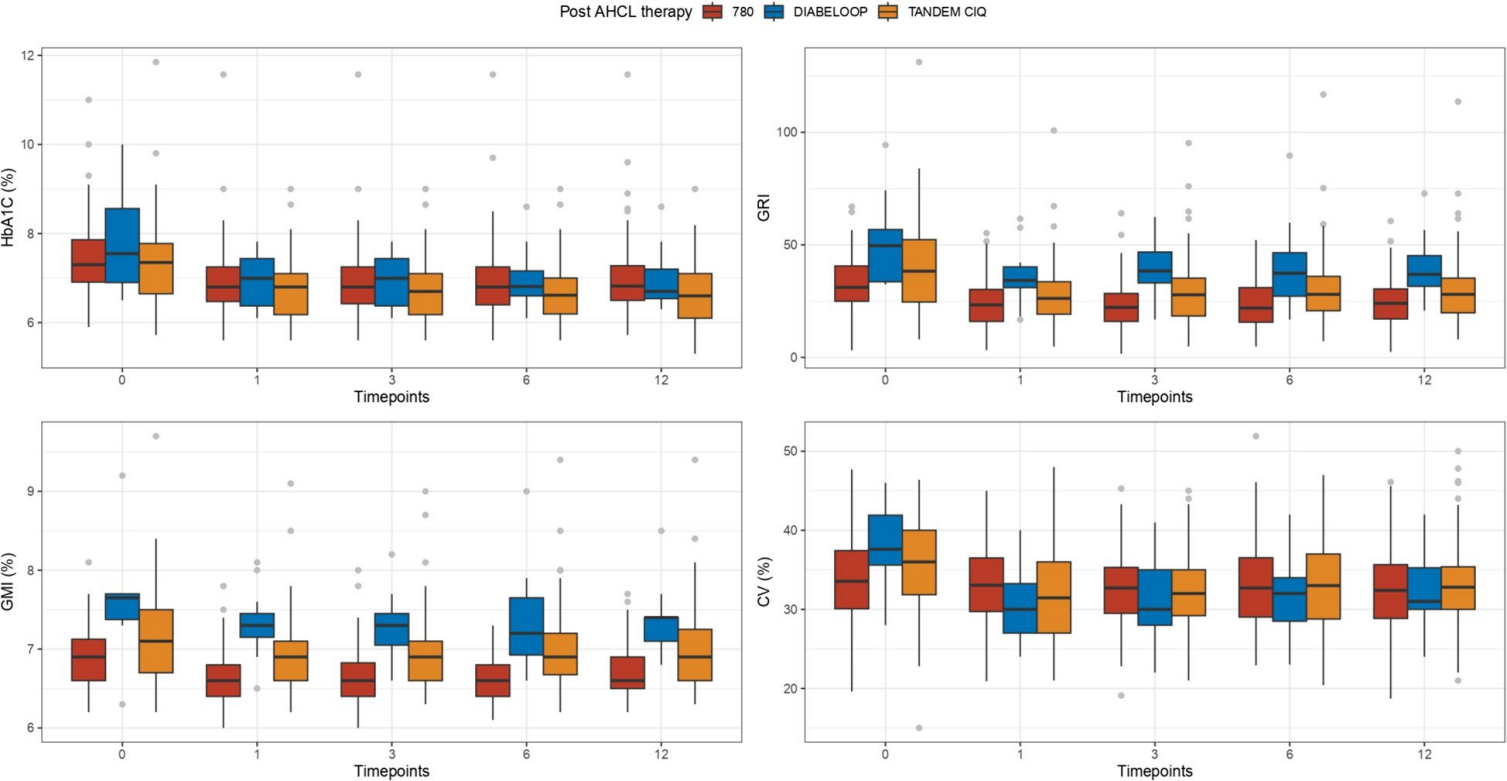
Boxplots showing the distribution of HbA1c, GRI, GMI, and CV at post-switch timepoints, stratified by AHCL therapy

**Fig. 4:**
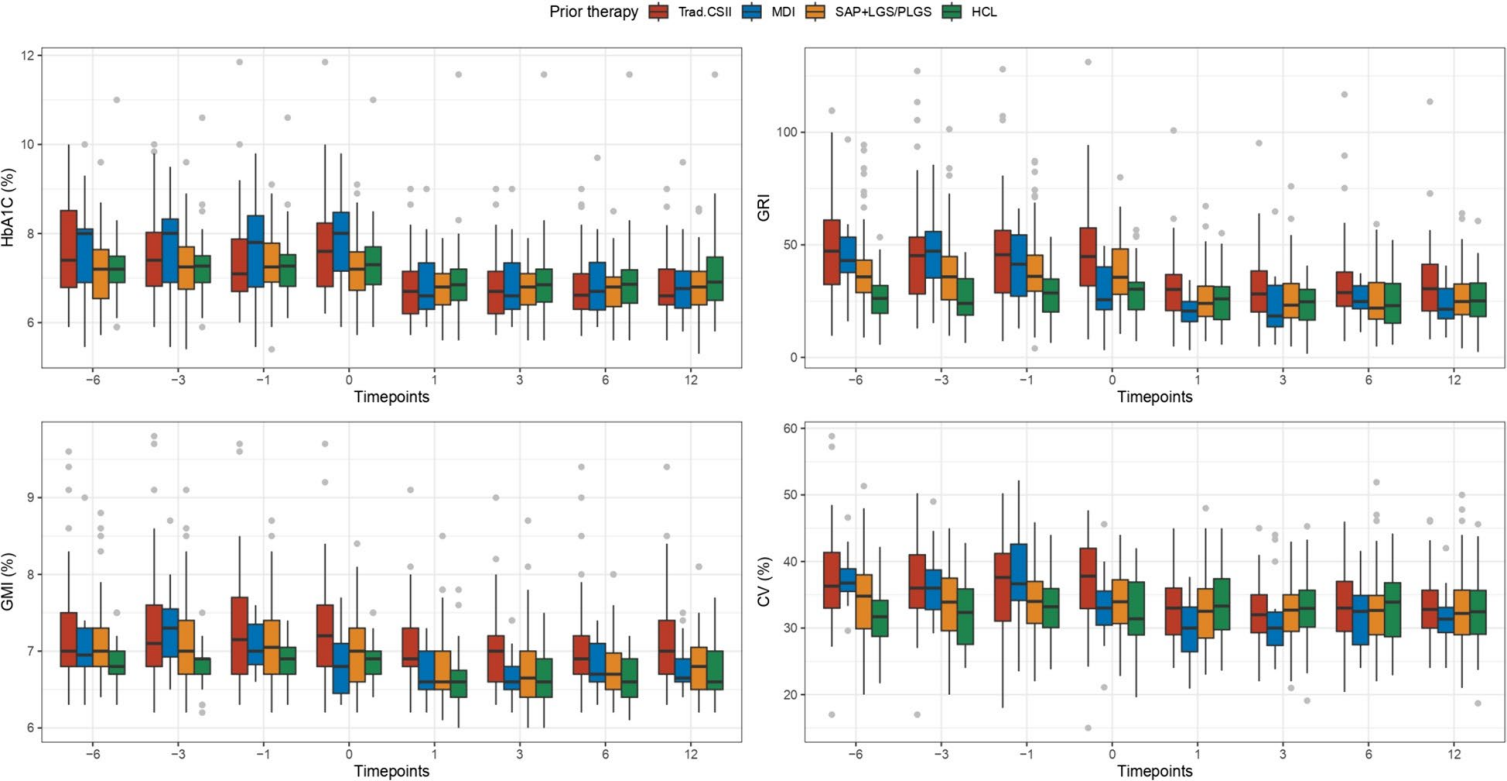
Boxplots showing the distribution of HbA1c, GRI, GMI, and CV, stratified by prior insulin therapy, over the entire study period

Figure 3 shows the distribution of HbA1c, GRI, GMI, and CV at post-switch timepoints, stratified by AHCL therapy. Figure 4 shows the distribution of these metrics over the entire study period, stratified by prior insulin therapy. Supplementary Tables S1 and S2 present mean values and standard deviations of HbA1c and CGM metrics at all time points, stratified by post-switch AHCL devices and by the four pre-switch insulin therapies, respectively.

### Complications

The evaluation of acute and chronic complications one year before and one year after the intervention was conducted. Acute complications included severe hypoglycemia and ketoacidosis, whereas chronic complications included arterial disease, nephropathy, neuropathy, retinopathy, and cardiopathy.

Acute complications were assessed both during the study period and throughout the entire available clinical history recorded at our hospital before the switch.

When comparing the 12 months pre- and 12 months post-change of therapy, no differences in acute complications (severe hypoglycemia 1 vs 1, ketoacidosis 1 vs 1) were noted. When the entire clinical history before the switch was compared with the one year following the switch, a notable decrease was observed in cases of severe hypoglycemia, dropping from 54 to 1 (p < 0.001), and in cases of ketoacidosis, reducing from 11 to 1 (p = 0.011). The singular case of severe hypoglycemia after the switch was linked to a sensor error, and the ketoacidosis incident to a pump cannula blockage, indicating no post-AHCL system activation complications were due to algorithm faults.

In terms of chronic complications, there was no significant change in the prevalence of arterial disease, nephropathy, neuropathy, retinopathy, or cardiopathy following the switch.

## Discussion

Our data demonstrated that the switch from different insulin therapies to AHCL systems led to a significant and early improvement in glycemic control. This improvement was consistently maintained over the 12-month observation period, without an increased risk of hypoglycemia and regardless of the device used.

This study evidenced for the first time a GRI reduction among type adult 1 diabetic patients using three different types of AHCL systems. While improvements in severe hypoglycemia and hyperglycemia are typically more pronounced in the GRI [5, 12], our cohort showed that GRI improvement was mainly driven by reductions in hyperglycemia and TIR, as revealed by our association analysis.

No correlation was found between GRI and TBR, this result might be due to both the excellent baseline values of TBR in our cohort and the protectivce role of AHCL against hypoglicemia. However, TBR_54-69_ improved after 3 months and this improvement persisted throughout the study period. The absence of significant variations in TBR_<54_ likely suggests that our study cohort was well educated and adept in managing insulin therapy. The Diabetology Department of Spedali Civili di Brescia has always supported therapeutic education in diabetic patients. The medical and nursing staff provide ongoing support for patients' educational needs, using structured educational tools, in line with the Italian SID-AMD guidelines. These guidelines highlight self-management education as a key aspect of diabetic care, offering a means to develop skills and abilities in managing the disease [22].

In particular, the highest quality educational approach is structured education, a program with a clear purpose and flexible content, designed to meet the clinical and psychological needs of the patient while adapting to their socio-cultural context [23].

The absence of significant gender differences in our cohort may be explained by the fact that with adequate education and competence in therapy management, all patients are capable of achieving excellent outcomes.

The increase in TIR and reduction in TAR represent expected results and are in line with those produced by experimental single-brand and observational clinical studies [24–26]. Specifically, our data showed that following the switch, the average TIR value increased from a suboptimal level to the therapeutic target (> 70%), and this improvement was consistently maintained for 12 months across all three AHCL systems.

BMI did not significantly change after the switch, which could be due to the fact that our cohort had a reduced number of obese patients, with the majority having normal or slightly elevated BMI values.

As regards the differences that emerged in our study from the comparison between the three AHCL, Diabeloop, significantly outperformed the others in terms of GRI, GMI, CV, TAR_>250,_ the Minimed™780G device recorded the highest average decrease in TAR_180-250_, while Tandem achieved the most significant reduction in TBR_54-69_ at T1. Beyond T1, the previously observed differences in performance improvements across the three devices were no longer evident. Diabeloop continued to demonstrate statistically significant better CV reductions at T3 and T6 and a significantly better TAR_>250_ at T3.

It is important to note that differences among the three AHCL systems were observed one month after the switch but then vanished over the study period. Therefore, attempting to understand the reasons behind these differences is highly speculative.

Given its inherent variability, it's logical that CV changed throughout the study period. However, the initial difference in metrics among the three AHCL systems raises intriguing points for discussion. It's plausible to suggest that the Diabeloop group’s superior GRI performance, could stem from nearly all patients (15 out of 16) transitioning from traditional pumps lacking automatic functions. Furthermore, Diabeloop's integration of the Model Predictive Controller (MPC) algorithm, enhanced with machine learning, enables its software to become self-learning through artificial intelligence analysis of the past two weeks' glucose data [27].

Regarding the patients using MiniMed 780G, 83 of the 99 patients made the transitioned from previous systems already equipped with an algorithm, although not as advanced, such as the Minimed 670G. These system could help patients control diabetes and manage the device better than traditional pumps. There was a routinary transition made in clinical practice, as these are devices successively placed on the market by the same manufacturer, therefore the overall improvement in metrics has been attenuated by the use of an algorithm. The greater reduction of TAR_180-250_ could be due to the algorithm of this system, that guarantees superior performance compared to its predecessors in controlling hyperglycemia [26].

Control-IQ group achieved the most significant reduction in TBR_54-69_ probably due to the algorithm of this system and that the glycemic target can be set in a range between 112.5 and 160 mg/dl [26].

Our study faced limitations, firstly its retrospective design, which inherently limits establishing causal relationships. Secondly, being a single-center cohort study limits the generalizability of our findings. Thirdly, the moderate sample size also affects the strength and reliability of our conclusions, especially when comparing metrics across different AHCL systems. In the statistical analysis used to compare the metrics of the three AHCL systems (based on multivariable mixed-effect regression models), we considered prior insuline therapy as a potential confounder. Including this variable in the models allowed us to obtain an adjusted estimate of the differences between the three groups. While this method does not completely eliminate the potential bias induced by previous therapy, it can substantially mitigate its effects.

Nonetheless, this study has notable strengths, including a long observational period, the inclusion of four distinct prior insulin therapies, and the comparison of three different AHCL systems.

Focusing on the GRI as the primary outcome represents a novel approach, due to its limited presence in scientific research and clinical practice. The GRI's potential for routine use and as a possible alternative to glycated hemoglobin is highlighted by its ability to reflect metabolic control. We analyzed our data to make a preliminary comparison of the various available devices. This analysis provides a foundation for future targeted studies aimed at answering specific clinical questions.

In conclusion, using an AHCL system leads to significant and sustained improvements in GRI and other metabolic control parameters for up to a year, independent of prior insulin therapies, baseline conditions, or device brands.

### Supplementary Information

Additional file 1. Figure S1A. Mean values with standard errors of HbA1c, GRI, GMI, CV, stratified by AHCL therapy, over the entire study period. S1**B** Mean values with standard errors of HbA1c, GRI, GMI, CV, stratified by prior insulin therapy, over the entire study period.

Additional file 2. Table S1. Mean values and standard deviations of HBA1c and CGM metrics at all the study timepoints, stratified by the three post-switch AHCL devices

Additional file 3. Table S2. Mean values and standard deviations of HBA1c and CGM metrics at all the study timepoints, stratified by the four pre-switch insulin therapies

## Data Availability

The data that support the findings of this study are available from the corresponding author, [ER], upon reasonable request.
