# Supplementary figures and images for: Impact on Glycemia Risk Index and other metrics in type 1 adult patients switching to Advanced Hybrid Closed-Loop systems: a one-year real-life experience
Source: Eur J Med Res. 2024 Jul 15;29:365. doi: 10.1186/s40001-024-01946-w (PMC11247841; doi:10.1186/s40001-024-01946-w)

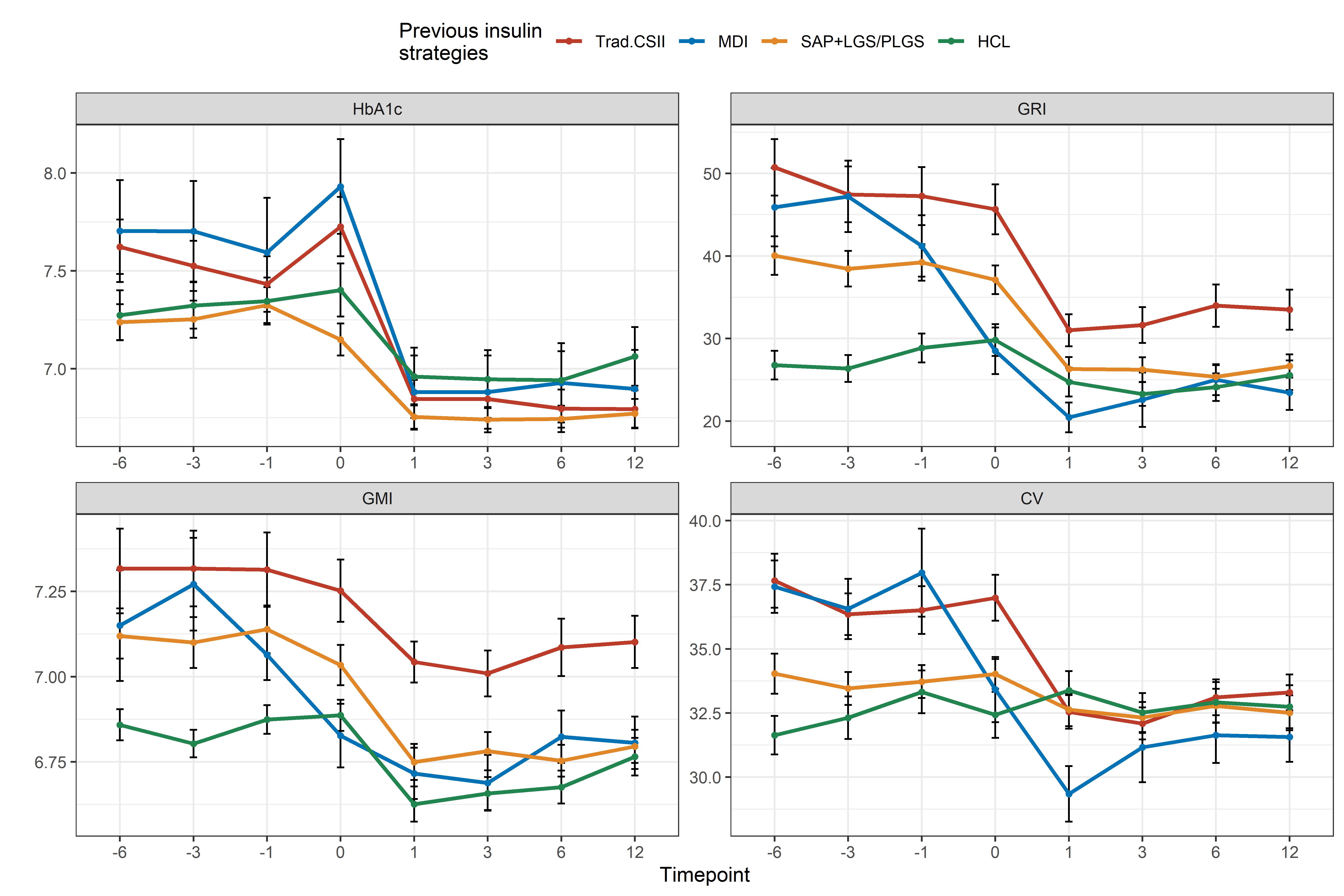

Supplement: Supplementary file 1 — Additional file 1. Figure S1A. Mean values with standard errors of HbA1c, GRI, GMI, CV, stratified by AHCL therapy, over the entire study period. S1B Mean values with standard errors of HbA1c, GRI, GMI, CV, stratified by prior insulin therapy, over the entire study period. [file 40001_2024_1946_MOESM1_ESM.jpeg]
