# Supplementary material for: Impact on Glycemia Risk Index and other metrics in type 1 adult patients switching to Advanced Hybrid Closed-Loop systems: a one-year real-life experience
Source: Eur J Med Res. 2024 Jul 15;29:365. doi: 10.1186/s40001-024-01946-w (PMC11247841; doi:10.1186/s40001-024-01946-w)
Supplement: Supplementary file 2 — Additional file 2. Table S1. Mean values and standard deviations of HBA1c and CGM metrics at all the study timepoints, stratified by the three post-switch AHCL devices [file 40001_2024_1946_MOESM2_ESM.docx]

**Supplementary Table 4.** Mean values and standard deviations of HBA1c and CGM metrics at all the study timepoints, stratified by the three post-switch AHCL devices

| Parameters | **T=-6** | **T=-3** | **T=-1** | **T=0** | **T=1** | **T=3** | **T=6** | **T=12** | **P** |
| --- | --- | --- | --- | --- | --- | --- | --- | --- | --- |
| **780 (N=99)** | | | | | | | | | |
| HbA1C (%) | 7.3±0.8 | 7.4±0.8 | 7.4±0.8 | 7.4±0.9 | 6.9±0.8 | 6.9±0.8 | 6.9±0.8 | 7±0.8 | <0.001 |
| GRI | 31.1±15.5 | 30.6±14.3 | 32.3±15.1 | 32.6±13.7 | 23.7±10.5 | 22.5±10.8 | 23.1±10.4 | 24±11 | <0.001 |
| GMI | 6.9±0.4 | 6.9±0.4 | 6.9±0.3 | 6.9±0.4 | 6.6±0.3 | 6.6±0.4 | 6.6±0.3 | 6.7±0.3 | <0.001 |
| CV | 33±5.3 | 32.8±4.7 | 34.1±5.7 | 33.6±5.6 | 33.1±4.9 | 32.4±4.8 | 33±5.2 | 32.3±5.3 | 0.2 |
| TAR_>250_ (%) | 5.3±6.6 | 5±5.3 | 6±5.7 | 5.1±4.7 | 2.6±3.2 | 2.9±3.8 | 2.7±2.8 | 3.4±3.8 | <0.001 |
| TAR_180-250_ (%) | 19.9±7.1 | 20.1±8 | 19.7±6.3 | 21.1±8.1 | 14±6 | 13.9±6.1 | 14.5±5.8 | 15.5±6.6 | <0.001 |
| TIR % | 72.2±12.3 | 72.3±12.1 | 71.6±11 | 70.8±11.5 | 80.1±8.4 | 80.5±8.9 | 79.9±8.1 | 78.7±9.4 | <0.001 |
| TBR_54-69_ (%) | 2.1±1.7 | 2±1.7 | 2.1±1.7 | 2.3±2.1 | 2.5±1.9 | 2.2±1.8 | 2.2±1.7 | 1.9±1.5 | 0.1 |
| TBR_<54_ (%) | 0.6±1.1 | 0.6±1.1 | 0.7±1 | 0.6±0.9 | 0.7±0.9 | 0.5±0.9 | 0.6±0.9 | 0.5±0.7 | 0.5 |
| HbA1c<7%, TIR>70%, TBR<4% | 18.5% | 18.5% | 10% | 8.3% | 39.3% | 42.9% | 39% | 43.9% | <0.001 |

P-values reported in the P column are obtained from the likelihood ratio test applied to the multivariable mixed-effects models for the comparison of mean values across all study time points.

| Parameters | **T=-6** | **T=-3** | **T=-1** | **T=0** | **T=1** | **T=3** | **T=6** | **T=12** | **P** |
| --- | --- | --- | --- | --- | --- | --- | --- | --- | --- |
| **DIABELOOP (N=16)** | | | | | | | | | |
| HbA1C (%) | 8.1±1.1 | 7.9±0.8 | 7.7±1 | 7.9±1.2 | 6.9±0.6 | 6.9±0.6 | 7±0.7 | 7±0.7 | <0.001 |
| GRI | 67.6±27.2 | 66.5±32.5 | 66.3±32.6 | 52.7±20.9 | 35.3±12.1 | 39.5±12.7 | 39.2±18.4 | 40.2±13.4 | <0.001 |
| GMI | 8±1.4 | 7.9±1.2 | 7.9±1.3 | 7.6±0.9 | 7.3±0.4 | 7.3±0.4 | 7.3±0.6 | 7.4±0.4 | 0.029 |
| CV | 43.1±7.6 | 39.2±5.4 | 39.3±6.3 | 37.9±5.9 | 30.8±4.6 | 31.1±5.1 | 31.7±5 | 32.1±4.8 | <0.001 |
| TAR_>250_ (%) | 19.2±21 | 24±18.9 | 23±18.2 | 17.2±13.5 | 8.7±7 | 9.6±6.8 | 10.6±10.5 | 10.6±7.5 | <0.001 |
| TAR_180-250_ (%) | 22.4±3 | 24.2±4.3 | 23.6±6.6 | 25.8±6.2 | 23.2±6.9 | 23.9±5.8 | 23.3±6.3 | 23.6±4.9 | 0.4 |
| TIR % | 51±18.5 | 48.5±18.5 | 49.4±21 | 55.2±15 | 67±9.8 | 64.6±9.3 | 64.7±13 | 64.1±9.6 | <0.001 |
| TBR_54-69_ (%) | 5.4±4.8 | 1.8±2.1 | 2.3±2.6 | 1.3±1.9 | 0.9±0.7 | 1.4±1.6 | 1±0.9 | 1.2±1.4 | <0.001 |
| TBR_<54_ (%) | 2±2.3 | 1.5±3.1 | 1.7±3.3 | 0.4±0.7 | 0.2±0.6 | 0.5±0.9 | 0.4±0.8 | 0.5±0.9 | 0.1 |
| HbA1c<7%, TIR>70%, TBR<4% | 0% | 0% | 16.7% | 0% | 20% | 10% | 35.7% | 33.3% | 0.2 |

P-values reported in the P column are obtained from the likelihood ratio test applied to the multivariable mixed-effects models for the comparison of mean values across all study time points.

| Parameters | **T=-6** | **T=-3** | **T=-1** | **T=0** | **T=1** | **T=3** | **T=6** | **T=12** | **P** |
| --- | --- | --- | --- | --- | --- | --- | --- | --- | --- |
| **TANDEM CIQ (N=83)** | | | | | | | | | |
| HbA1C (%) | 7.4±1 | 7.3±1 | 7.3±1 | 7.4±1 | 6.7±0.7 | 6.7±0.7 | 6.7±0.7 | 6.7±0.7 | <0.001 |
| GRI | 46.1±22.5 | 44.6±21.2 | 43.5±21.5 | 40.7±20.6 | 28.8±14.9 | 29.3±15.6 | 30.5±16.5 | 30.7±16.6 | <0.001 |
| GMI | 7.2±0.7 | 7.2±0.7 | 7.2±0.7 | 7.1±0.6 | 7±0.5 | 6.9±0.5 | 7±0.5 | 7±0.5 | <0.001 |
| CV | 35.6±7.7 | 35.4±6.6 | 35.1±7 | 35.1±6.4 | 32±5.8 | 32.1±5.5 | 32.8±5.7 | 33.2±5.8 | <0.001 |
| TAR_>250_ (%) | 10.2±9.6 | 10.6±11.1 | 10.5±10.5 | 10.1±11.8 | 5.7±6.9 | 5.8±6.9 | 6.3±7.8 | 6.9±8.1 | <0.001 |
| TAR_180-250_ (%) | 25.5±11.3 | 25±11 | 24.5±11.3 | 22.9±9.9 | 18.9±8.2 | 18.8±8.6 | 19.2±8.9 | 19±8.2 | <0.001 |
| TIR % | 60.6±18.7 | 61.4±17.6 | 62.1±18.3 | 64.1±18.6 | 73.5±12.7 | 73.4±13.2 | 72.5±14.4 | 72.3±13.8 | <0.001 |
| TBR_54-69_ (%) | 2.9±2.8 | 2.4±2.2 | 2.3±2.1 | 2.4±2.1 | 1.5±1.3 | 1.5±1.5 | 1.6±1.5 | 1.4±1.4 | <0.001 |
| TBR_<54_ (%) | 0.8±2.3 | 0.6±1.1 | 0.5±0.9 | 0.5±0.9 | 0.3±0.5 | 0.4±0.7 | 0.3±0.6 | 0.4±0.6 | 0.023 |
| HbA1c<7%, TIR>70%, TBR<4% | 18.8% | 14.8% | 25.5% | 19.2% | 47.7% | 43.5% | 48.5% | 45.5% | <0.001 |

P-values reported in the P column are obtained from the likelihood ratio test applied to the multivariable mixed-effects models for the comparison of mean values across all study time points.
