# Supplementary material for: Impact on Glycemia Risk Index and other metrics in type 1 adult patients switching to Advanced Hybrid Closed-Loop systems: a one-year real-life experience
Source: Eur J Med Res. 2024 Jul 15;29:365. doi: 10.1186/s40001-024-01946-w (PMC11247841; doi:10.1186/s40001-024-01946-w)
Supplement: Supplementary file 3 — Additional file 3. Table S2. Mean values and standard deviations of HBA1c and CGM metrics at all the study timepoints, stratified by the four pre-switch insulin therapies [file 40001_2024_1946_MOESM3_ESM.docx]

**Supplementary Table 5.** Mean values and standard deviations of HBA1c and CGM metrics at all the study timepoints, stratified by the four pre-switch insulin therapies

| Parameters | **T=-6** | **T=-3** | **T=-1** | **T=0** | **T=1** | **T=3** | **T=6** | **T=12** | **P** |
| --- | --- | --- | --- | --- | --- | --- | --- | --- | --- |
| **Traditional CSII with associated sensor (N=59)** | | | | | | | | | |
| HbA1C (%) | 7.6±1.1 | 7.5±1 | 7.4±1.1 | 7.7±1.2 | 6.8±0.8 | 6.8±0.8 | 6.8±0.7 | 6.8±0.7 | <0.001 |
| GRI | 50.7±26.2 | 47.5±26 | 47.3±27.1 | 45.7±23.2 | 31±14.9 | 31.6±16.7 | 34±19.7 | 33.5±18.7 | <0.001 |
| GMI | 7.3±0.9 | 7.3±0.9 | 7.3±0.8 | 7.3±0.7 | 7±0.5 | 7±0.5 | 7.1±0.6 | 7.1±0.6 | <0.001 |
| CV | 37.7±8.1 | 36.4±6.2 | 36.5±7.2 | 37±6.9 | 32.5±5 | 32.1±4.8 | 33.1±5.4 | 33.3±5.3 | <0.001 |
| TAR_>250_ (%) | 12.5±14.2 | 12.9±14.4 | 13.5±14.1 | 12.5±12.5 | 6.9±7.4 | 7.1±7.7 | 8.3±10.1 | 8.5±9.6 | <0.001 |
| TAR_180-250_ (%) | 23.2±9.4 | 23.5±8.3 | 22.5±8.7 | 23.3±7.8 | 19.8±6.7 | 19.2±7.6 | 19.7±8 | 19.4±7.2 | <0.001 |
| TIR % | 59.5±20 | 60.5±18.4 | 61±19.8 | 61.4±17.8 | 71.6±11.9 | 71.7±13.5 | 70.1±15.8 | 70.4±14.7 | <0.001 |
| TBR_54-69_ (%) | 3.8±3.3 | 2.3±1.9 | 2.4±2.3 | 2.3±1.9 | 1.3±1.1 | 1.6±1.4 | 1.6±1.4 | 1.4±1.3 | <0.001 |
| TBR_<54_ (%) | 1.1±2 | 0.8±1.7 | 0.7±1.7 | 0.5±0.7 | 0.3±0.6 | 0.4±0.7 | 0.4±0.8 | 0.4±0.7 | 0.013 |
| HbA1c<7%, TIR>70%, TBR<4% | 16.1% | 15.4% | 26.5% | 13.2% | 37.8% | 37.2% | 47.9% | 41.3% | <0.001 |

P-values reported in the P column are obtained from the likelihood ratio test applied to the multivariable mixed-effects models for the comparison of mean values across all study time points.

| Parameters | **T=-6** | **T=-3** | **T=-1** | **T=0** | **T=1** | **T=3** | **T=6** | **T=12** | **P** |
| --- | --- | --- | --- | --- | --- | --- | --- | --- | --- |
| **MDI (N=20)** | | | | | | | | | |
| HbA1C (%) | 7.7±1.2 | 7.7±1.2 | 7.6±1.2 | 7.9±1.1 | 6.9±0.8 | 6.9±0.8 | 6.9±0.9 | 6.9±0.9 | <0.001 |
| GRI | 45.9±21.2 | 47.2±19.4 | 41.2±16.7 | 28.5±12.6 | 20.4±8 | 22.6±14.7 | 25±8.4 | 23.4±9.3 | <0.001 |
| GMI | 7.2±0.7 | 7.3±0.6 | 7.1±0.3 | 6.8±0.4 | 6.7±0.3 | 6.7±0.4 | 6.8±0.3 | 6.8±0.3 | <0.001 |
| CV | 37.4±4.6 | 36.6±5.3 | 38±7.7 | 33.4±5.7 | 29.3±4.9 | 31.2±6.1 | 31.6±4.9 | 31.6±4.3 | <0.001 |
| TAR_>250_ (%) | 9.5±11.7 | 10.3±9.6 | 8.2±6.4 | 7.4±14.6 | 2.4±2.2 | 3.1±3.7 | 3.5±3.2 | 3.7±3.4 | <0.001 |
| TAR_180-250_ (%) | 23.8±8.8 | 27±11.6 | 22.9±10.4 | 19.2±9 | 15.1±6.8 | 14.4±6.9 | 18.1±7 | 16.7±6.3 | <0.001 |
| TIR % | 62.1±16.8 | 59.1±15.6 | 64.9±12.4 | 70.8±21.1 | 80.7±8.1 | 80.1±10.8 | 76.5±8.2 | 77.9±8.9 | <0.001 |
| TBR_54-69_ (%) | 3.6±3.5 | 2.9±2.6 | 3.4±2.4 | 2.4±2.2 | 1.5±1.1 | 1.9±2.4 | 1.6±1.7 | 1.2±1.1 | 0.049 |
| TBR_<54_ (%) | 1±1.3 | 0.7±1 | 0.5±0.7 | 0.2±0.5 | 0.3±0.6 | 0.5±1.1 | 35.3% | 38.9% | 0.4 |
| HbA1c<7%, TIR>70%, TBR<4% | 25% | 15.4% | 33.3% | 20% | 60% | 64.3% | 35.3% | 58.8% | 0.050 |

P-values reported in the P column are obtained from the likelihood ratio test applied to the multivariable mixed-effects models for the comparison of mean values across all study time points.

| Parameters | **T=-6** | **T=-3** | **T=-1** | **T=0** | **T=1** | **T=3** | **T=6** | **T=12** | **P** |
| --- | --- | --- | --- | --- | --- | --- | --- | --- | --- |
| **SAP plus LGS and PLGS (N=75)** | | | | | | | | | |
| HbA1C (%) | 7.2±0.8 | 7.3±0.8 | 7.3±0.8 | 7.1±0.7 | 6.8±0.6 | 6.7±0.6 | 6.7±0.6 | 6.8±0.7 | <0.001 |
| GRI | 40±20.2 | 38.5±18.7 | 39.2±19.2 | 37.1±15.1 | 26.3±12.5 | 26.2±13 | 25.4±12.1 | 26.7±12.2 | <0.001 |
| GMI | 7.1±0.6 | 7.1±0.6 | 7.1±0.6 | 7±0.5 | 6.7±0.5 | 6.8±0.5 | 6.8±0.4 | 6.8±0.4 | <0.001 |
| CV | 34±6.8 | 33.5±5.5 | 33.7±5.6 | 34±5.1 | 32.6±5.5 | 32.3±5.2 | 32.8±5.7 | 32.5±5.9 | 0.028 |
| TAR_>250_ (%) | 7.8±7.6 | 7.8±8.9 | 8.5±8.2 | 6.7±6 | 3.9±5.2 | 4.2±5.6 | 3.8±4.3 | 4.5±4.6 | <0.001 |
| TAR_180-250_ (%) | 24.5±10.7 | 23.6±11.2 | 23.9±10.5 | 23±10.1 | 16±8.6 | 16.6±8.7 | 16.3±8.2 | 17.1±8.5 | <0.001 |
| TIR % | 64.6±16.8 | 65.8±16.8 | 65.1±16.7 | 67.2±13.9 | 77.2±11.4 | 76.8±11.8 | 77.4±11.1 | 76.1±11.2 | <0.001 |
| TBR_54-69_ (%) | 2.3±2.2 | 2.1±2 | 1.9±1.6 | 2.5±2.3 | 2.2±1.7 | 1.9±1.6 | 1.9±1.6 | 1.8±1.7 | 0.04 |
| TBR_<54_ (%) | 0.8±2.3 | 0.6±1.1 | 0.6±0.9 | 0.7±1 | 0.6±0.8 | 0.5±0.9 | 0.5±0.8 | 0.5±0.7 | 0.5 |
| HbA1c<7%, TIR>70%, TBR<4% | 11.6% | 13.7% | 12.8% | 9.8% | 43.5% | 41.4% | 43.8% | 50% | <0.001 |

P-values reported in the P column are obtained from the likelihood ratio test applied to the multivariable mixed-effects models for the comparison of mean values across all study time points.

| Parameters | **T=-6** | **T=-3** | **T=-1** | **T=0** | **T=1** | **T=3** | **T=6** | **T=12** | **P** |
| --- | --- | --- | --- | --- | --- | --- | --- | --- | --- |
| **HCL (N=44)** | | | | | | | | | |
| HbA1C (%) | 7.3±0.8 | 7.3±0.8 | 7.3±0.8 | 7.4±0.9 | 7±1 | 6.9±1 | 6.9±1 | 7.1±1 | <0.001 |
| GRI | 26.8±11.5 | 26.4±10.9 | 28.9±11.6 | 29.8±12.8 | 24.7±11.6 | 23.3±9.6 | 24.1±11.1 | 25.6±12 | <0.001 |
| GMI | 6.9±0.3 | 6.8±0.3 | 6.9±0.3 | 6.9±0.3 | 6.6±0.3 | 6.7±0.3 | 6.7±0.3 | 6.8±0.4 | <0.001 |
| CV | 31.6±5 | 32.3±5.5 | 33.3±5.5 | 32.4±5.9 | 33.4±5 | 32.5±5 | 32.9±5.2 | 32.7±5.6 | 0.3 |
| TAR_>250_ (%) | 4.5±4.6 | 4±3.1 | 4.7±3.9 | 4.7±4.6 | 2.8±3.3 | 3±2.5 | 2.9±2.9 | 3.9±3.9 | <0.001 |
| TAR_180-250_ (%) | 18.2±5.9 | 17.9±5.2 | 18.4±5.8 | 20.4±7.4 | 14.8±6.5 | 15.2±6.6 | 15.3±6.5 | 16.6±7 | <0.001 |
| TIR % | 75.3±9.6 | 75.9±8.2 | 74.3±8.8 | 72.6±10.5 | 79.1±9.2 | 79.3±8.3 | 78.9±8.9 | 77.2±10.3 | <0.001 |
| TBR_54-69_ (%) | 1.6±1.3 | 1.7±1.4 | 1.9±1.5 | 1.8±1.7 | 2.6±2.1 | 2.1±1.9 | 2.2±1.7 | 1.9±1.5 | 0.011 |
| TBR_<54_ (%) | 0.4±0.6 | 0.5±0.9 | 0.6±1 | 0.6±1 | 0.7±0.9 | 0.5±0.8 | 0.6±0.9 | 0.5±0.7 | 0.3 |
| HbA1c<7%, TIR>70%, TBR<4% | 25% | 19.4% | 7.1% | 14.3% | 35.1% | 35.3% | 37.1% | 29.7% | 0.061 |

P-values reported in the P column are obtained from the likelihood ratio test applied to the multivariable mixed-effects models for the comparison of mean values across all study time points.
